# Evaluation of C-Reactive Protein in Patients with Chronic Obstructive Pulmonary Disease

**DOI:** 10.3889/oamjms.2015.061

**Published:** 2015-05-27

**Authors:** Ljiljana Simonovska, Irfan Ahmeti, Vladimir Mitreski

**Affiliations:** 1 *Institute for Lung Diseases and Tuberculosis, Faculty of Medicine, Ss Cyril and Methodius University of Skopje, Skopje, Republic of Macedonia*; 2 *University Clinic of Endocrinology, Diabetes and Metabolic Disorders, Faculty of Medicine, Ss Cyril and Methodius University of Skopje, Skopje, Republic of Macedonia*

**Keywords:** C-reactive protein, COPD, bronchial obstruction, co-morbidites, proinflammatory cytokines

## Abstract

**BACKGROUND:**

Chronic Obstructive Pulmonary Disease (COPD) is associated with evidence of systemic oxidative stress, activation of circulating inflammatory cells and increased plasma level of proinflamatory cytokines which include C-reactive protein (CRP). CRP is one biomarker of extrapulmonary or systemic consequences of COPD that can be detected.

**AIM:**

The aim of this research is to determine whether the level of CRP statistically significantly correlates with the level of bronchial obstruction and the accompanying co-morbidities in patients with COPD.

**MATERIAL AND METHODS:**

This study included 80 patients with exacerbation of COPD, hospitalised at the Institute for Lung Diseases and Tuberculosis in Skopje. We measured the level of CRP in the blood in all of these patients in fasting conditions. The classification of COPD patients by the severity of airflow limitation was made according to the actual version of the Global initiative for chronic Obstructive Lung Disease (GOLD). The Student’s Independent Samples t-test was used for the statistic analysis of the data.

**RESULTS:**

In 52 (65%) of the patients with exacerbation of COPD we detected an increase of the mean value of CRP. The statistical analysis using the Student’s t-test showed statistically significant differences in the mean value of CRP in patients with different level of bronchial obstruction. Hypertension, heart failure, diabetes mellitus, hyperlipidemia, coronary disease, and CVI were confirmed as co-morbidities in 45 (73.1%) of the patients, hypertension being the most frequent one (40%). The statistical analysis using the Student’s t-test showed statistically significant difference of the mean value of CRP (p< 0.01) depending on the number of co-morbidities.

**CONCLUSION:**

In 52 (65%) of the patients with exacerbation of COPD, were detected an increase of the mean value of CRP. The mean values of CRP statistically significantly correlate with the level of bronchial obstruction and the number of co-morbidities in patients with COPD.

## Introduction

Chronic Obstructive Pulmonary Disease (COPD) is a major cause of chronic morbidity and mortality throughout the world [1]. It is primarily characterized by the presence of airflow limitation resulting from airways inflammation and remodelling often associated with parenchymal destruction and development of emphysema [2].

Increasing evidence suggests that COPD is a complex disease involving more than airflow obstruction [1].

In many patients the disease is associated with several systemic manifestations that can effectively result in impaired functional capacity, worsening dyspnea, and reduced health-related quality of life [2, 3]. The most common manifestations include the presence of concomitant cardiovascular compromise, diabetes, malnutrition involving primary the loss and dysfunction of skeletal muscles, osteoporosis, clinical depression and anxiety [3-6]. Currently, the GOLD definition of COPD includes the words “some significant extrapulmonary effects that may contribute to the severity in individual patients”. It has suggested that COPD should be renamed a “chronic systemic inflammatory syndrome” [7, 8]. COPD is associated with evidence of systemic oxidative stress, activation of circulating inflammatory cells and increased plasma level of proinflamatory cytokines which include C-reactive protein (CRP), IL-6, fibrinogen, leucocytes and TNF [3, 5, 7, 9].

Patients with COPD have higher circulating levels of IL-6 [2, 7]. This cytokine is a potent stimulator of CRP production by the liver and may account for the increase in circulating CRP found in patients with COPD [2]. It is an acute-phase protein synthesized predominantly by the hepatocytes in response to tissue damage or inflammation reflecting the total systemic burden of inflammation of individuals [5]. CRP is one biomarker of extrapulmonary or systemic consequences of COPD that can be detected clinically and that could also be measured.

The aim of this research is to determine whether the level of CRP in patients with COPD statistically significantly correlates with the level of bronchial obstruction and the accompanying co-morbidities.

## Material and Methods

This study included 80 patients with exacerbation of COPD, hospitalised at the Institute for Lung Diseases and Tuberculosis. Out of these 80 patients, 48 were male and 32 were female. We measured the level of CRP in the blood in all of these patients in fasting conditions.

The classification of COPD patients by the severity of airflow limitation was made according to the actual version of the Global initiative for chronic Obstructive Lung Disease (GOLD) i.e. GOLD 1, GOLD 2, GOLD 3 and GOLD 4 (mild, moderate, severe, very severe airways obstruction) [8].

**Table 1:** Measurement of forced expiratory volume in one second (FEV1)

Group	Subjects	Obstruction	FEV1
GOLD1	13	mild	FEV1. 80% predicted
GOLD2	20	moderate	50%.FEV1< 80% predicted
GOLD3	32	severe	30.FEV1< 50%
GOLD4	15	very severe	FEV1. 30% predicted or
			FEV1<50% predicted plus
			chronic respiratory failure

The Student’s Independent Samples t-test was used for the statistic analysis of the data.

## Results

The mean values of CRP in patients with different level of bronchial obstruction are shown in Table 2.

**Table 2:** Mean values of CRP in patients with COPD

Group	N (number)	Mean value of CRP mg/l ± SD	t-test	p
GOLD1	13	8.6 ± 5.5	GOLD1/GOLD2 = 2.3	<0.05
GOLD2	20	17.1 ± 9.8	GOLD1/GOLD3 = 4.9	<0.01
GOLD3	32	36.7 ± 27.6	GOLD1/GOLD4 = 6.5	<0.01
GOLD4	15	77.0 ± 38.1	GOLD3/GOLD4 = 3.6	<0.01

Student’s Independent Samples t-test

The statistical analysis conducted using the Student’s t-test showed significant statistical differences in the mean values of CRP in patients with different level of bronchial obstruction. The highest mean value of CRP was registered in patients with very severe bronchial obstruction (GOLD 4).

**Figure 1:**
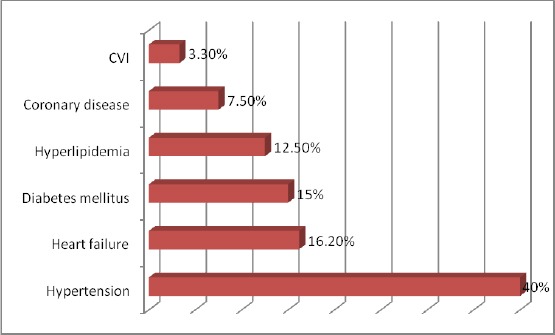
*Distribution of co-morbidities*.

The frequency and distribution of co-morbidities in patients with COPD is shown in Figure 1.

The mean values of CRP in correlation with the number of co-morbidities are shown in Table 3.

**Table 3:** Mean value of CRP according to the number of co-morbidities

Comorbidities	N (number)	Mean value of CRP mg/l ± SD	t-test	p
COPD	25	11.6 ± 6.3	COPD/COPD+CoMb1=3.29	<0,01
COPD+CoMb1	20	26.2 ± 17.8	COPD/COPD+CoMb2=4.42	<0.01
COPD+CoMb2	20	40.8 ± 31.0	COPD/COPD+CoMb3=4.72	<0.01
COPD+CoMb3	11	71.4 ± 41.0	COPD/COPD+CoMb4=5.96	<0.01
COPD+CoMb4	4	92.5 ± 26.8		

The Student’s Independent Samples t-test; COPD +CoMb1 - COPD plus one comorbidity; COPD+Co Mb2 – COPD plus two comoprbidities; COPD+Co Mb3 – COPD plus three comoprbidities; COPD+Co Mb4 – COPD plus four comoprbidities.

The statistical analysis conducted using the Student’s t-test showed statistically significant increase of the mean value of CRP (p< 0.01) in correlation with the number of co-morbidities.

## Discussion

COPD is a complex disease involving more than airflow obstruction [1]. COPD is associated with evidence of systemic oxidative stress, activation of circulating inflammatory cells and increased plasma level of proinflamatory cytokines. Individuals with COPD had significantly raised levels of several markers of inflammation which include C-reactive protein (CRP), IL-6, fibrinogen, leucocytes and TNF [2-5, 7, 9, 10]. Our retrospective study also registers increase of the mean value of CRP in 52 (65 %) of the patients.

Rennard and his associates [2] reported on the relationship between FEV1 and levels of various systemic inflammatory markers such as CRP. This analysis indicated that reduced lung function significantly correlated with the elevated levels of systemic inflammatory markers.

Nillawar and his associates [10], in their study carried out with 45 patients with COPD, point out that there is statistically significant increase of the value of CRP, whose mean value of FEV 1 was 45.27 % +/-15%. In our study, in 52 (65%) of the patients with exacerbation of COPD, were detected an increase of the mean value of CRP.

The statistical analysis of the mean values of CRP using the Student’s t-test showed statistically significant increase of the mean values of CRP in correlation with the level of bronchial obstruction (P < 0.05; P < 0.01).

In many patients, COPD is associated with several systemic manifestations that can effectively result in impaired functional capacity, worsening dyspnoea, and reduced health-related quality of life [2, 3]. In our study, hypertension, heart failure, diabetes mellitus, hyperlipidaemia, coronary disease, CVI were confirmed as co-morbidities in 45 (73.1%) of the patients, hypertension being the most frequent one (40%).

Several studies have demonstrated a strong relationship between COPD and cardiovascular disease with COPD patients having a two-fold increase in risk for morbidity and mortality due to a cardiovascular disease [5, 10, 11]. The underlying mechanism is still not fully understood. However, COPD is recognized as a systemic inflammation which might extend beyond the lungs causing other co-morbidities. Consequently, the inflammatory state in patients with COPD might lead to the development of atherosclerosis, which is also known as an inflammatory process. It is thought that the inflammation observed in COPD patients plays a significant role in the pathogenesis of atherosclerosis. Furthermore, in patients with both COPD and cardiovascular disease, increasing C-reactive protein levels are present, which confirms the presence of a systemic inflammation [12]. The relationship between COPD, systemic inflammation and a cardiovascular disease is of particular importance, since more than one half of all patients with COPD die due to cardiovascular causes.

Rutten and his associates [13] point out that 20% of the patients with COPD had a non-diagnosed left-sided heart failure. Our study also demonstrates 23% of left-sided heart failure in patients with COPD.

Leonardo and his associates [14] indicate that 20% of the patients had left-sided heart weakness, and 50% of the patients had one or more components of metabolic syndrome. According to data, the prevalence of diabetes in COPD varies from 2% (Sidney [15]), 12% (Mapel and associates [16]) to 16% (Wash and Thomasov [17]). In our study, we registered hyperlipidemia in 12.5%, and diabetes in 15% of the patients. The systematic inflammation accounts for the connection between COPD and diabetes mellitus type 2. The diabetes is independently associated with reduction of the lung function, and the frequently common obesity may additionally deteriorate the clinical picture in patients with COPD. Recent studies suggest that raised level of CRP, IL 6 and TNF result in alteration of the metabolic processes and resistance to insulin.

In our study, too, the statistical analysis conducted with the Student’s t-test, demonstrated statistically significant increase of the mean value of CRP (p < 0.01) depending on the number of co-morbidities.

In conclusion, in 52 (65%) of the patients with exacerbation of COPD, were detected an increase of the mean value of CRP. The mean values of CRP statistically significantly correlate with the level of bronchial obstruction and the number of co-morbidities in patients with COPD.
